# Establishment and validation of a three-dimensional finite element model for degenerative lumbar scoliosis

**DOI:** 10.3389/fbioe.2025.1669961

**Published:** 2025-11-27

**Authors:** Wei Guo, Wanzhong Yang, Jie Yang, Xin Zhao, Honglai Zhang, Zemin Wang, Shiyong Wang, Rong Ma, Zhaohui Ge

**Affiliations:** 1 Department of Orthopedics, General Hospital of Ningxia Medical University, Yinchuan, China; 2 The First Clinical Medical College, Ningxia Medical University, Yinchuan, China

**Keywords:** degenerative lumbar scoliosis, finite element model, model validation, grid sensitivity analysis, biomechanical effects

## Abstract

**Objective:**

The objective of this study was to construct a three-dimensional finite element model of degenerative lumbar scoliosis (DLS) and validate its effectiveness, providing a reliable theoretical tool for optimizing surgical plans and biomechanical research.

**Methods:**

A 3D finite element model (FEM) of Lenke-Silva type IV DLS was constructed from patient CT data using Mimics, Geomagic Warp, SolidWorks, and ANSYS, incorporating cortical bone, cancellous bone, and intervertebral discs with defined material properties and contact relationships. Geometric validation was performed by comparing vertebral alignment and offset with radiographic measurements, while biomechanical validation involved applying a 400N axial load and 7.5 Nm torque (flexion/extension, lateral bending, and axial rotation) to L1 and comparing the results with established literature data.

**Results:**

The successfully constructed L1-S1 DLS finite element model comprised 1,255,696 tetrahedral elements (1.5 mm mesh size) and 1,919,710 nodes. Geometric validation demonstrated excellent agreement with radiographic measurements, showing <1 error in Cobb and lumbar lordosis, and <1.76 mm deviation in vertebral centroid alignment. Biomechanical validation revealed that the segmental range of motion (ROM) at L2-3 through L4-5 under 7.5 Nm loading conditions (flexion/extension, lateral bending, and axial rotation) matched established literature data, confirming model reliability.

**Conclusion:**

The DLS three-dimensional finite element model constructed in this study exhibits high anatomical fidelity and biomechanical reliability, enabling dynamic simulation of spinal mechanical behavior under complex loads, thereby providing an experimental foundation for surgical plan optimization and complication prediction.

## 1 Introduction

Degenerative lumbar scoliosis (DLS) is a three-dimensional spinal deformity that arises from progressive disc and facet-joint degeneration, leading to coronal and sagittal imbalance, axial rotation, and intractable pain (Birknes et al., 2008; Liu et al., 2009; Jimbo et al., 2012; Hou et al., 2022). Its prevalence increases markedly after the sixth decade and now represents one of the most complex entities encountered by spine surgeons, physiatrists, physiotherapists and chiropractic and traditional-Chinese-medicine practitioners alike (Wiczenbach et al., 2023). While conservative care—including supervised physiotherapy, chiropractic manipulation, acupuncture and pharmacological protocols—can mitigate early symptoms and delay progression, a substantial proportion of patients ultimately require surgery because of intractable pain, neurological compromise or structural instability. The pathogenesis of DLS has not yet been fully elucidated, and the selection of surgical options (such as fusion segments and fixation methods) remains controversial. There is an urgent need to provide objective evidence through biomechanical studies (Chu, 2022; Chu et al., 2023). The aetiology of DLS remains incompletely understood, and the choice of fusion levels, osteotomy grade and fixation strategy continues to generate controversy (Katz et al., 2019). High-quality biomechanical evidence that links individual radiographic phenotype to surgical outcome is therefore urgently needed. Cadaveric and animal experiments, however, are hampered by scarce specimens, irreproducible loading conditions and ethical constraints, and they cannot replicate the dynamic muscle-controlled environment of the living human spine (Stirling et al., 2014). Finite-element (FE) modelling offers a non-invasive, repeatable platform to quantify internal stresses and segmental kinematics under physiological loading, but its value depends on anatomical fidelity, appropriate material laws and rigorous validation—steps that are frequently incomplete in existing DLS models (Zheng et al., 2015).

We therefore constructed and experimentally validated a patient-specific three-dimensional FE model of Lenke–Silva type IV DLS. A 21° Cobb angle represents the most prevalent moderate stage of DLS, filling a critical gap unaddressed by existing mild-scoliosis models and offering high clinical relevance. The study aims are: (i) to create an anatomically accurate L1–S1 FE model from high-resolution CT data, incorporating grade-II/III disc degeneration and facet arthrosis; and (ii) to verify its geometric and biomechanical validity against radiographic measurements and published ROM, intradiscal pressure and facet-force data. The converged model will provide a clinically credible platform for comparing different surgical strategies and implant configurations in future multicentre studies.

## 2 Materials and methods

### 2.1 Experimental equipment and software information

The Philips computed tomography CT scanner (GE, Discovery CT750HD) was provided by the Department of Radiology at the General Hospital of Ningxia Medical University. Scan data: slice thickness 0.625 mm, image matrix set at 512 × 512. Computer and processing software information: computer configuration: Windows 10 × 64-bit operating system, Intel(R) Core (TM) i7-3700 processor, 1T solid-state drive, 32 GB memory. Mimics 20.0 3D reconstruction software (Materalise,Inc.,Belgium) and 3-Matic 12.0 modeling software (Materalise,Inc.,Belgium). Geomagic Studio 2021 reverse engineering software (Geomagic, Inc., United States). SolidWorks 2018 CAD software (Dassault, Inc., France). ANSYS18.0 finite element analysis software (Ansys, Inc., United States).

### 2.2 Experimental methods and procedures

#### 2.2.1 Acquisition of raw CT data

This study was approved by the Research Ethics Committee of the General Hospital of Ningxia Medical University (Ethics Approval Number: KYLL-2022-1137), and the participating patients signed informed consent forms. The CT data of a 67-year-old female patient with Lenke type 4 DLS (weight: 63 kg, height: 162 cm) from the Department of Orthopedics at the General Hospital of Ningxia Medical University were saved in Digital Imaging and Communications in Medicine (DICOM) format. The patient had no history of spinal infections, spinal trauma, spinal tumors, or congenital spinal deformities, and the imaging findings were consistent with the anatomical characteristics of the normal population.

#### 2.2.2 Extract the three-dimensional model of the lumbosacral spine (L1-S1)

**FIGURE 1:**
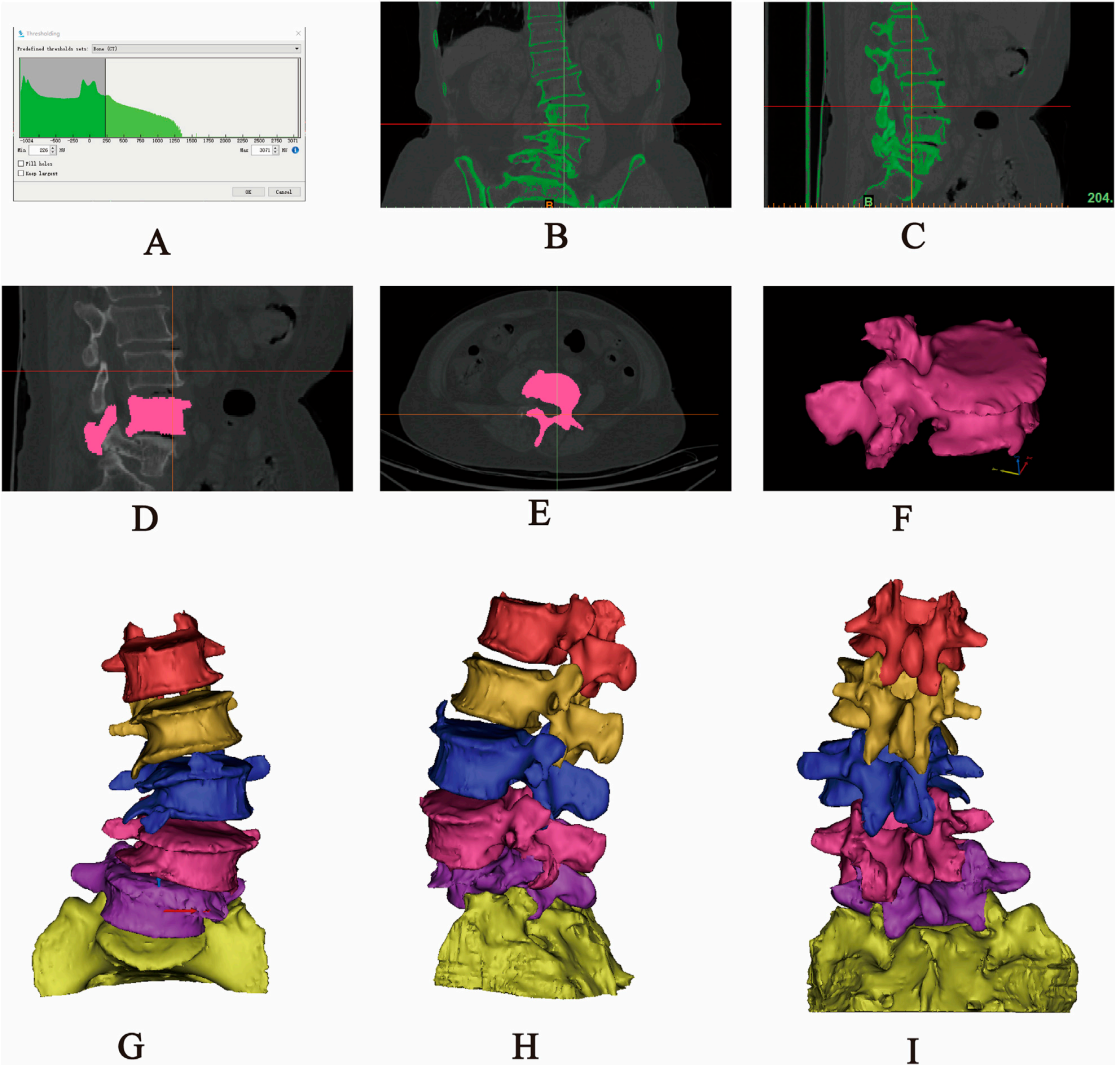
Extraction of the L1-S1 model in Mimics software. **(A)** Threshold segmentation. **(B–C)** Region growing. **(D–F)** Mask editing. **(G)** Front view. **(H)** Lateral view. **(I)** Rear view.

The original CT data in DICOM format were imported into Mimics 20.0 software. A grayscale threshold range of 226–3017 HU was applied to segment bone from surrounding soft tissue. Using the Split Mask function, the L1–S1 vertebral mask was isolated. The grayscale values were then fine-tuned to enhance bone tissue clarity. Subsequently, the Region Grow tool was employed to remove unconnected anatomical structures, including adjacent soft tissues, organs, and extraneous bone segments, ensuring that only the L1–S1 region remained. Finally, a new and refined mask was generated, focusing solely on the target vertebra. Within the generated mask, the Edit Masks function was selected, and the Draw tool was applied layer by layer across all views to meticulously segment the target vertebra from adjacent anatomical structures, including the zygapophyseal joints, osteophytes, ribs, and other bony attachments. Missing regions within the target vertebra were then filled using the Draw function to ensure structural continuity. Following 2D mask segmentation and refinement, the Region Grow tool was reapplied, and the Calculate Part command was executed to generate a complete 3D model of the isolated vertebral body. The model underwent preliminary smoothing using the Smooth function (Figure 1). After sequentially extracting all vertebrae (L1–S1) through this process, a preliminary three-dimensional finite element model of the lumbar spine was obtained. Each vertebral model was individually exported and saved in STL format for further analysis.

#### 2.2.3 Optimization processing model (L1-S1)

The lumbosacral spine 3D bone model obtained from Mimics software, despite having undergone smooth processing, still exhibits numerous rough and uneven areas on its surface, including irregular osteophyte proliferation, holes, protrusions, and sharp regions. These defects will lead to an increase in the number of elements in subsequent analyses, thereby increasing the complexity and error rate of computational analysis. As a professional reverse engineering software, Geomagic Wrap 2021 can efficiently reconstruct 3D geometric models into solid models through reverse engineering. Its core advantages are reflected in: achieving rapid modeling based on intelligent point cloud data integration technology, supporting high-precision NURBS surface modeling to restore complex anatomical forms, equipping with adaptive mesh generation algorithms to optimize the topology of polygonal models, and ensuring the editability and transferability of model data in the engineering chain through seamless compatibility with mainstream CAD platforms (such as SolidWorks). These characteristics make it one of the preferred tools for model surface reconstruction in orthopedic biomechanics research. Therefore, the STL format file of a single vertebral body needs to be imported into the reverse engineering software Geomagic Wrap 2021 for further optimization to improve the accuracy of the model. Since the mesh size is closely related to the smoothness of the vertebral bone, an excessively large mesh will result in lower model quality, while an excessively small mesh will lead to increased computational load and reduced efficiency. After importing the STL file, the mesh size needs to be reset in the polygon command. Based on the actual conditions of this study, the mesh size is set to 1 mm, and the red boundaries are deleted. Subsequently, select the “Mesh Doctor” command to diagnose and analyze the model, smoothing out holes, depressions, protrusions, and spikes on the surface of the vertebra until all results from the “Mesh Doctor” check are “0.” Then, using surface optimization operations such as “Remove Features,” “Delete Spikes,” “Fill Holes,” “Sandpaper,” “Quick Smooth,” and “Reduce Noise” to gradually smooth out the uneven areas of the model surface. After repeating the above operations, select the “Mesh Doctor” command again to perform a comprehensive check on the model. When all results are “0,” proceed with the surfacing operation to obtain a single vertebral geometric model with a smooth surface, reasonable structure, and clear contours. (2) Constructing Precise Surface Patch Models: After completing the aforementioned smoothing process, click on the “Precise Surface” interface to perform the surfacing operation. Sequentially select the operation buttons “Detect Contour Lines,” “Generate Contour Lines,” “Construct Surface Patches,” “Construct Grids,” and “Fit Surface” to generate relatively regular quadrilateral surface patches. In this study, the surface patch count is manually set to “500,” and then the number of surfaces is appropriately adjusted according to the actual requirements and aesthetic considerations of the research. During the aforementioned operations, select the “Edit Contour Lines” command to add, adjust, or delete contour lines, and select “Repair Surface Patches” to appropriately adjust the intersecting surface patches, irregular quadrilateral surface patch angles, or height corner points present in the generated surface patches. After verification, proceed with constructing the grid and fitting the surface. Subsequently, the cancellous bone model was created. The fitted model was duplicated, and after duplication, the interface was switched to “Polygon.” The “Offset” command was selected to perform a “Global” inward offset of 1 mm on the model. Structures such as the pedicle, spinous process, transverse process, and articular process were selected and deleted, retaining only the vertebral body portion of the vertebra. Then, the “Hole Filling” command was chosen to repair the voids created by the removal, ultimately obtaining the cancellous bone model (Guo et al., 2020; Wang et al., 2021; Zhang et al., 2022). The aforementioned operations for the cortical bone model were repeated, resulting in the final fitted cancellous bone model (Supplementary Figure S1). Finally, the cortical bone model and cancellous bone model were saved in “STP” format and imported into SolidWorks 2018 for model assembly.

#### 2.2.4 Assemble the L1-S1 lumbar spine 3D model

**FIGURE 2:**
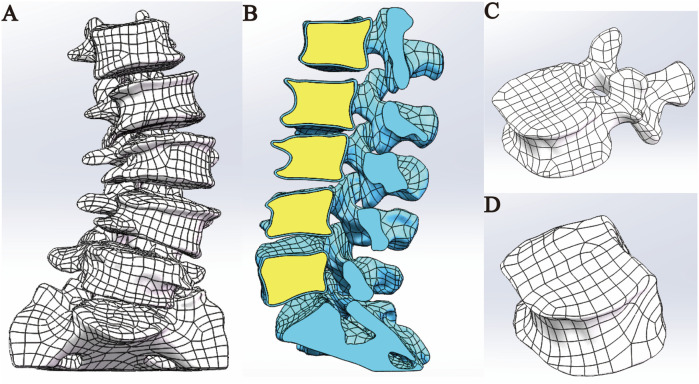
Model assembly. **(A)** Overall model. **(B)** Sectional view. **(C)** Cortical bone model. **(D)** Cancellous bone model.

**FIGURE 3:**
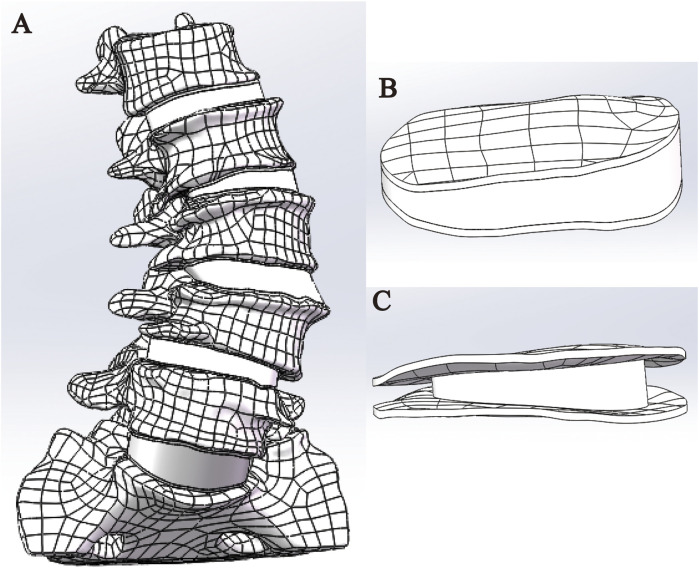
Disc assembly. **(A)** Modeling of intervertebral disc; **(B)** nucleus pulposus; **(C)** endplates.

The optimized L1–S1 vertebral models in STP format were imported into SolidWorks 2018 software, where they were properly assembled into a complete lumbosacral spine structure. Subsequently, key anatomical components—including intervertebral discs (with distinct nucleus pulposus and annulus fibrosus regions), cartilaginous endplates, and articular cartilage—were incorporated into the model. This step ensured that the reconstructed spinal segment closely replicated the physiological characteristics of natural human anatomy. The specific procedural workflow was as follows: (1) Each vertebral model in STP format was individually imported into SolidWorks 2018, where initial geometry validation was performed. Following successful import verification, the models were saved as native part files (*.SLDPRT) to enable subsequent editing, precise anatomical assembly, and necessary geometric refinements during later modeling stages. (2) Assembly Creation: A new assembly file was created, and each vertebral part file was sequentially imported into the assembly workspace. The L1 vertebra was designated as a fixed component, while all remaining vertebrae were set to a floating configuration. Using the Mate tool, all components were precisely aligned at the global origin to establish proper anatomical relationships. Following assembly completion, an Interference Check was executed to identify geometric conflicts. If interference was detected, the model was transferred back to Geomagic Wrap 2021 for targeted geometry modification until all conflicts were resolved. The finalized interference-free assembly was then exported and saved as a consolidated part file for subsequent use. (3) Cortical and Cancellous Bone Structure Construction: The assembly model in part format was opened to initiate bone layer differentiation. The cortical bone components of L1-L5 were temporarily hidden using the Hide function. The cancellous bone entities of L1-L5 were then duplicated through the Move/Copy Entities command. Using the Combine tool with the Subtract operation, the duplicated cancellous structures were subtracted from the complete vertebral bodies, generating anatomically accurate cortical bone shells that perfectly conform to their respective cancellous cores. This process yielded complete lumbar vertebral models with distinct cortical and cancellous bone layers (Figure 2). (4) Construction of Intervertebral Disc, Cartilage, and Endplate: All vertebral models were set to visible mode, and the L2 vertebra was isolated via right-click context menu. Three reference points were selected on the superior surface of L2 to establish Reference Plane 1, upon which a 2D sketch was created. Using the Spline Curve tool, the general outline of the intervertebral disc was traced along the vertebral body’s superior contour, with subsequent adjustments available for precise anatomical matching. After completing the sketch, a two-sided symmetric extrusion was performed using the Extrude Boss feature (with ‘Do Not Merge Results’ enabled), creating a preliminary disc structure that maintained contact with adjacent vertebrae. The extruded volume was then temporarily hidden to facilitate surface operations. Through the Offset Surface command (0 mm spacing), exact replicas of the L2 superior surface (Offset Surface 1) and L1 inferior surface (Offset Surface 2) were generated, with extents slightly exceeding the disc boundaries. These surfaces served as splitting tools in a Direct Editing operation, trimming the extruded boss to create a precisely fitted disc model. A secondary offset operation (1 mm inward spacing) produced Offset Surfaces 3 and 4, which, when applied through additional splitting, divided the structure into three distinct anatomical components: the central intervertebral disc with superior and inferior cartilaginous endplates (Wang et al., 2021). However, given the anatomical complexity of human intervertebral discs-which consist of distinct nucleus pulposus and annulus fibrosus components-additional segmentation was required. The isolated intervertebral disc model was selected, and Reference Plane 2 was created on its surface. Using the Spline Curve tool, the nucleus pulposus geometry was carefully sketched according to physiological proportions (occupying 50%–60% of the total disc volume). After exiting sketch mode, the Split command was executed using this sketch as the cutting tool, dividing the intervertebral disc model while preserving both entities to yield separate nucleus pulposus and annulus fibrosus components (Liu et al., 2017; Lu and Lu, 2019; Ke et al., 2023). This refined modeling process was systematically repeated to create all lumbar disc structures (including nucleus pulposus, annulus fibrosus, and superior/inferior endplates) for the L2-3, L3-4, L4-5, and L5-S1 segments (Figure 3). (5) Construction of Articular Cartilage: The lumbar zygapophyseal (facet) joints, forming the posterior elements of the spinal three-joint complex, serve as fundamental biomechanical components in the vertebral motion segment. These synovial joints maintain three-dimensional spinal stability through two primary mechanisms: (1) physiological load transmission via precisely congruent articular surfaces, and (2) kinematic constraint of excessive vertebral displacement through geometric articulation. Their functional significance becomes particularly evident during spinal extension and axial rotation, where they critically modulate segmental motion patterns by regulating contact stress distribution across articular surfaces (Huang et al., 2016). The articular cartilage was modeled using a methodology analogous to endplate construction. A reference plane was established on the superior articular process surface, positioned contralateral to the corresponding inferior articular process. The cartilage profile was then sketched and extruded using the Boss feature. The articular surfaces of both superior and inferior processes were selected as zero-offset (0 mm) reference surfaces for subsequent splitting operations, which precisely trimmed the extruded volume to create anatomically accurate cartilage geometry. Upon completion of all components, the final L1-S1 degenerative lumbar scoliosis (DLS) model was exported in x_t format for subsequent finite element analysis (Supplementary Figure S2).

#### 2.2.5 Adding material properties

##### 2.2.5.1 Establishing a material library

Before proceeding to the next step of finite element analysis, it is necessary to assign corresponding material properties to each structure of the full lumbosacral spine model (Table 1). The models established above are imported into ANSYS 18.0 software in x_t format files, and a Static Structural analysis project is created. Based on the research results in the references, the material properties of cortical bone, cancellous bone, endplate, annulus fibrosus, nucleus pulposus, and articular cartilage are set (Polikeit et al., 2003; Lu and Lu, 2019; Ling et al., 2021; Y et al., 2022). The degeneration parameters were assigned to the degenerated intervertebral discs (L2-3, L3-4, L4-5) (Guo et al., 2020). In this study, the static finite element analysis method was employed; thus, only the two core mechanical parameters of each structural material, namely, the elastic modulus and Poisson’s ratio, needed to be specified (Shirazi-Adl et al., 1986). These two parameters (elastic modulus and Poisson’s ratio) quantitatively characterize a material’s physical and mechanical behavior under varying stress states. Higher values of elastic modulus correspond to greater stiffness and load-bearing capacity, while elevated Poisson’s ratios indicate increased resistance to volumetric deformation. Conversely, lower values of these parameters reflect materials with reduced structural strength but enhanced compliance and deformation adaptability (Shirazi-Adl et al., 1986). In addition, all structures of the lumbar spine model in this study are assumed to be homogeneous, continuous, and isotropic linear elastic materials (Chevalier et al., 2021).

**TABLE 1:** Material properties of the lumbar spine finite element model.

Material	Elastic modulus (MPa)	Poisson’s ratio	Stiffness (N/mm)
Cortical bone	12,000	0.3	-
Cancellous bone	100	0.2	-
Endplate	2000	0.2	-
Annulus fibrosus (healthy)	4.2	0.45	-
Annulus fibrosus (degenerated)	8.4	0.45	
Nucleus pulposus (healthy)	1	0.499	-
Nucleus pulposus (degenerated)	8.4	0.45	
Articular cartilage	25	0.25	-
Ligaments			
Anterior longitudinal ligament (ALL)	7.8	-	8.74
Posterior longitudinal ligament (PLL)	10	-	5.83
Supraspinous ligament	8	-	15.38
Interspinous ligament	10	-	0.19
Ligamentum flavum	15	-	15.75
Intertransverse ligament	10	-	2.39

##### 2.2.5.2 Adding ligaments

In the human body, the movement of the spine is not only related to the skeletal structure but also closely associated with the ligaments surrounding the spine. Therefore, selecting the correct anatomical positions to add the corresponding ligaments in the model ensures that the model more accurately reflects the real conditions of spinal movement. Add the Anterior Longitudinal Ligament (ALL), Posterior Longitudinal Ligament (PLL), Supraspinous Ligament (SSL), Interspinous Ligament (ISL), Ligamentum Flavum (LF), and Intertransverse Ligament (ITL) to the corresponding anatomical locations on the model based on anatomical positioning. In this study, all ligaments were simulated using only Spring linear elements that are subjected to tension but not compression. The Spring element is a type of nonlinear spring element defined to bear only tension and cannot be compressed, and its constraint magnitude is represented by defining the stiffness value (N/mm) (Du et al., 2016).

#### 2.2.6 Grid sensitivity analysis

In this study, the Mesh module of Ansys 18.0 software was utilized to perform unstructured meshing on the L1-S1 lumbar spine 3D model. The density of the finite element mesh is closely related to the simulation accuracy; the higher the density, the closer the simulation results are to the actual situation, but the computational load and required time also increase significantly. The purpose of mesh sensitivity analysis is to find an appropriate mesh resolution that includes a sufficiently large number of elements to ensure the accuracy of the simulation. Simultaneously, the total number of elements at the expected mesh resolution should be as small as possible to save simulation time. Therefore, it is necessary to improve computational efficiency while ensuring computational accuracy (Xu et al., 2017a). The study by Ayturk et al. (2010) demonstrated that in finite element models, the axial rotation of the lumbar spine is most sensitive to mesh density. When the difference in calculation results between two consecutive mesh densities is less than 5%, the mesh can be considered convergent, and the mesh density at this point can meet the computational accuracy requirements (Ayturk and Puttlitz, 2011). This study constructed three L1-S1 lumbar spine models with different mesh sizes, namely, Mesh1 (1 mm), Mesh2 (1.5 mm), and Mesh3 (2 mm). A 7.5N axial torque was applied to the superior surface of the L1 vertebral body in the three lumbar spine models with different mesh sizes, and the maximum Von Mises stresses in the cortical bone, cancellous bone, articular cartilage, endplate, and intervertebral disc were compared.

#### 2.2.7 Set boundary conditions, loads, and contact relationships

This study utilized the Mechanical APDL module of Ansys 18.0 software to define the boundary conditions and loads of the model. Based on the biomechanical characteristics of the lumbar spine, the bottom surface of the sacrum was fully fixed (constraining all degrees of freedom) to simulate the stability of the pelvis during physiological activities (Patwardhan et al., 1999). A vertical downward load of 400N (the weight of the upper body of the patient, approximately two-thirds of the body weight) and a torque of 7.5 Nm in different directions (flexion, extension, left lateral bending, right lateral bending, left rotation, right rotation) were applied to the upper surface of the L1 cortical bone (Yamamoto et al., 1989; Liu et al., 2017). In this study, a pure moment of 7.5 Nm was applied in multiple directions, based on the standardized loading conditions widely adopted in the literature to simulate the physiological range of motion (ROM) of the human lumbar spine. This magnitude has been validated by numerous *in vitro* experiments and finite element studies as a non-destructive load representative of normal physiological activities, making it suitable for model validation and biomechanical comparative analyses.

In setting up the contact relationships, it is determined based on the relative positions of the parts of the lumbar model and whether slippage occurs. For areas that are in contact but do not experience slippage (such as the interface between cortical bone and cancellous bone, the interface between nucleus pulposus and annulus fibrosus, the interface between vertebral body and endplate, and the interface between endplate and annulus fibrosus), a “Bonded” contact relationship is set. For areas that are in contact but do not undergo relative movement (such as the interface between articular cartilage and vertebral body), a “No Separation” contact type is established (Shirazi-Adl et al., 1984; Xu et al., 2017b; Wang et al., 2018).

### 2.3 Validate the effectiveness of the lumbar spine model

#### 2.3.1 Comparison with X-ray images

On the superior surface of the L1 vertebral body in the established L1-S1 lumbar spine model, a vertical load of 400N and a torsional load of 7.5 N·m were applied (Liu et al., 2017) to simulate flexion and extension movements. Subsequently, measurements and observations were compared with actual lumbar spine anteroposterior and lateral radiographs, as well as hyperextension and hyperflexion radiographs. Cobb angle and lumbar lordosis (LL) were measured, respectively, to verify their geometric similarity.

#### 2.3.2 Centroid measurement method of the vertebral body

In the Surgimap software, the patient’s preoperative lumbar spine anteroposterior radiograph was imported for anatomical landmark identification. Four corner points were manually marked on each vertebral body from L1 to L5. Two diagonal lines were then constructed by connecting opposing corner point pairs, with their intersection defining the geometric center of each respective vertebral body. Subsequently, the midpoint of the S1 superior endplate border was identified, from which a vertical line perpendicular to the true horizontal plane (parallel to the radiographic baseline) was drawn to establish the Center Sacral Vertical Line (CSVL) as the reference axis for spinal alignment assessment (Cho et al., 2014). The perpendicular distances between the Center Sacral Vertical Line (CSVL) and the geometric center of each vertebral body (L1 through L5) were quantitatively measured and documented. For the degenerative lumbar scoliosis (DLS) model, these measurements were performed using SolidWorks’ measurement module, which precisely replicates the radiographic measurement methodology described previously while accounting for three-dimensional anatomical variations in the digital model.

#### 2.3.3 Stress loading verification

This study compares the ROM results from the *in vitro* experiments by Yamamoto et al. (1989) and the finite element analysis by Liu et al. (2017). A 400 N vertical compressive load was applied perpendicular to the superior endplate of the L1 vertebral body, while the S1 inferior surface was fully constrained. Six pure moment loading conditions (7.5 N·m each) were sequentially imposed to simulate physiological motions: flexion, extension, left/right lateral bending, and left/right axial rotation. The resulting ROM at the L2-L3, L3-L4, and L4-L5 motion segments was computationally derived through finite element analysis. This study employs a novel command flow technique to perform three-dimensional dynamic measurements of lumbar ROM. The innovation of this method lies in establishing virtual remote marker points on the vertebral surface, utilizing the numerical computation module within finite element analysis software to execute preset command flows, thereby capturing the trajectory parameters of these marker points in spatial motion in real-time (Supplementary Figure S3). The system specifically employs a spatial coordinate transformation algorithm to perform matrix operations on collected radian values and displacement quantities, calculating the angular movements of marker points about the three orthogonal axes (X, Y, and Z). Unlike traditional two-dimensional screenshot measurement methods limited to single-plane data acquisition, this method utilizes three-dimensional motion decomposition technology to simultaneously quantify spinal ROM in the sagittal, coronal, and horizontal planes, while employing vector synthesis formulas to compute composite motion parameters. Experimental verification demonstrates that this automated measurement scheme not only significantly improves data collection efficiency but also, through its three-dimensional spatial resolution characteristics, maintains measurement errors within ±0.5° effectively circumventing the inherent information loss associated with two-dimensional projection methods.

## 3 Results

### 3.1 Establish a three-dimensional finite element model of DLS

**FIGURE 4:**
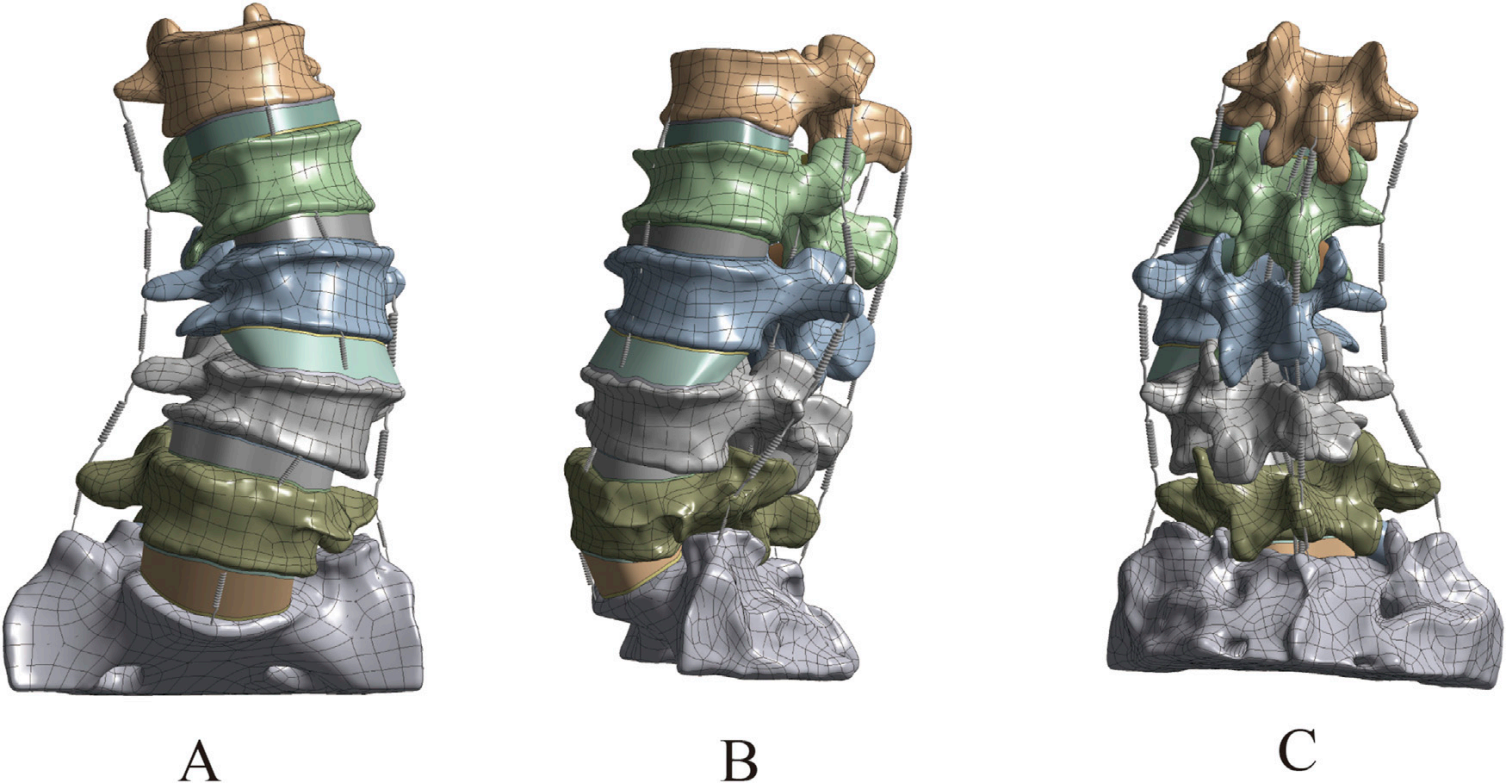
Complete 3D finite element model of DLS. **(A)** Front view; **(B)** Lateral view; **(C)** Rear view.

This study successfully constructed a three-dimensional finite element model of the DLS L1-S1 segment, including five lumbar vertebrae, one sacral vertebra, five intervertebral discs, ten endplates, ten articular cartilages, and seven types of ligaments (Figure 4).

### 3.2 The grid sensitivity analysis results of the DLS model

**FIGURE 5:**
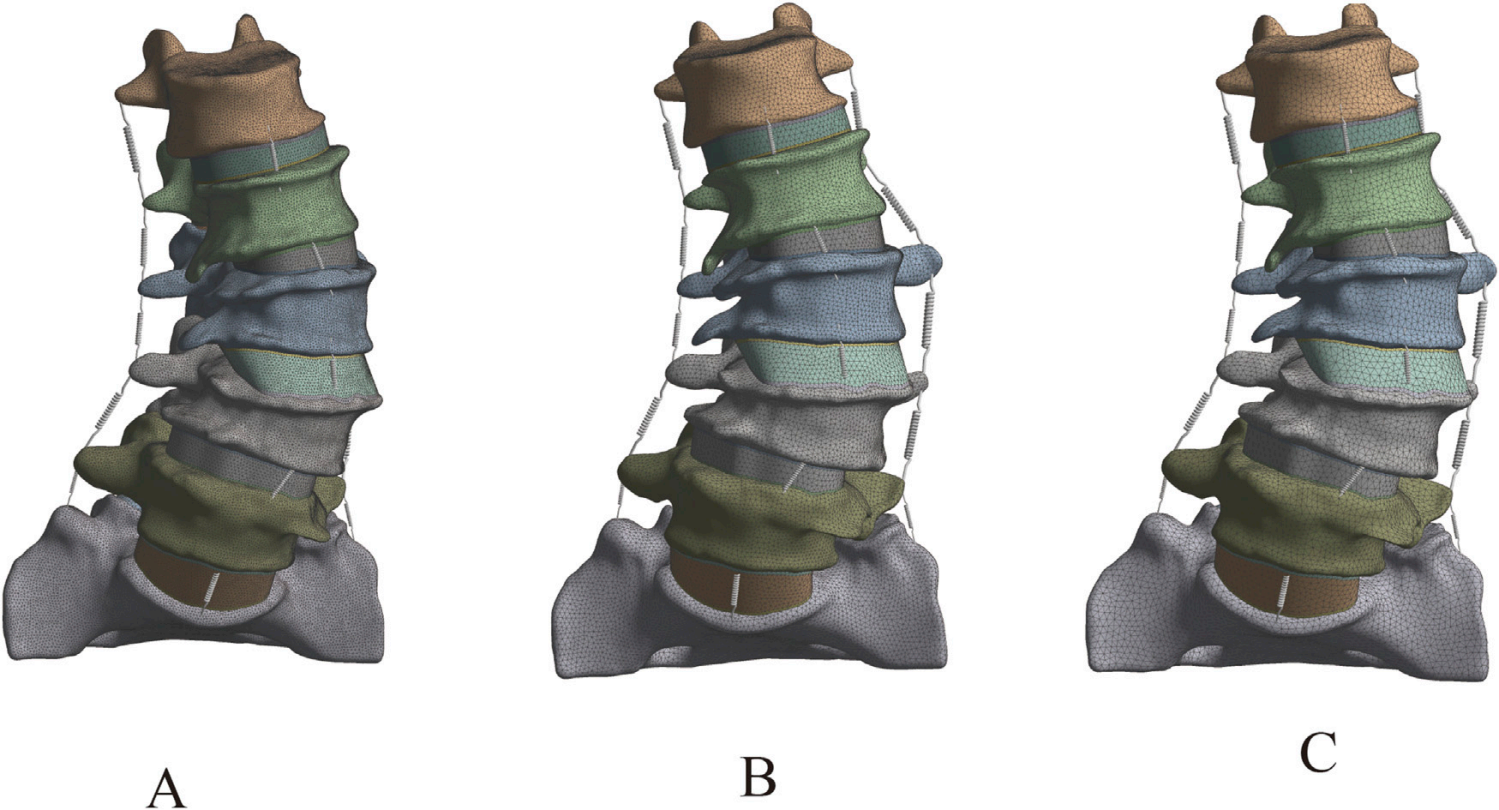
DLS models with different mesh densities: **(A)** Mesh1, **(B)** Mesh2, **(C)** Mesh3.

The lumbar spine models with three different mesh sizes (Mesh1, Mesh2, Mesh3) were subjected to axial loading, and the convergence criterion was determined based on the maximum Von Mises stress in cortical bone, cancellous bone, intervertebral disc, endplate, and articular cartilage. Mesh1, Mesh2, and Mesh3 comprised 4,006,484, 1,255,696, and 583,552 tetrahedral elements, and 5,854,205, 1,919,710, and 92,369 nodes, respectively (Figure 5). The computation times on the same computer were 68 h, 16 h, and 6 h, respectively. The maximum difference in von Mises stress between Mesh1 and Mesh3 (13.4%) occurred on the endplate. The minimum difference in von Mises stress between Mesh1 and Mesh3, indicating the lowest sensitivity to endplate mesh resolution (6.8%), was observed in the intervertebral disc, while the difference between Mesh2 and Mesh3 was 3.3%. In all structures of the DLS model, the differences in Von Mises stress between Mesh1 and Mesh2 were less than 5% (Supplementary Figure S4; Table 2). Based on the comparison of computational time and results, and considering factors such as time cost, this study ultimately set the mesh size of the lumbar spine finite element model to 1.5 mm, resulting in a total of 1,255,696 tetrahedral elements and 1,919,710 nodes in the DLS finite element model.

**TABLE 2:** The grid sensitivity analysis (MPa).

Von Mises	Mesh1	Mesh2 (vs. Mesh1, %)	Mesh3 (vs. Mesh1, %)
Cortical bones	4.787	4.6088 (3.9%)	4.2541 (12.5%)
Cancellous bone	1.769	1.6899 (4.7%)	1.629 (8.6%)
Cartilaginous endplate	2.0656	1.995 (4.8%)	1.9336 (13.4%)
Intervertebral disc	5.7885	5.5245 (3.5%)	5.1041 (6.8%)
Facet	5.2245	5.05 (3.5%)	5.05 (11.5%)

### 3.3 Validation results of the DLS three-dimensional finite element model

#### 3.3.1 In comparison with X-rays

**FIGURE 6:**
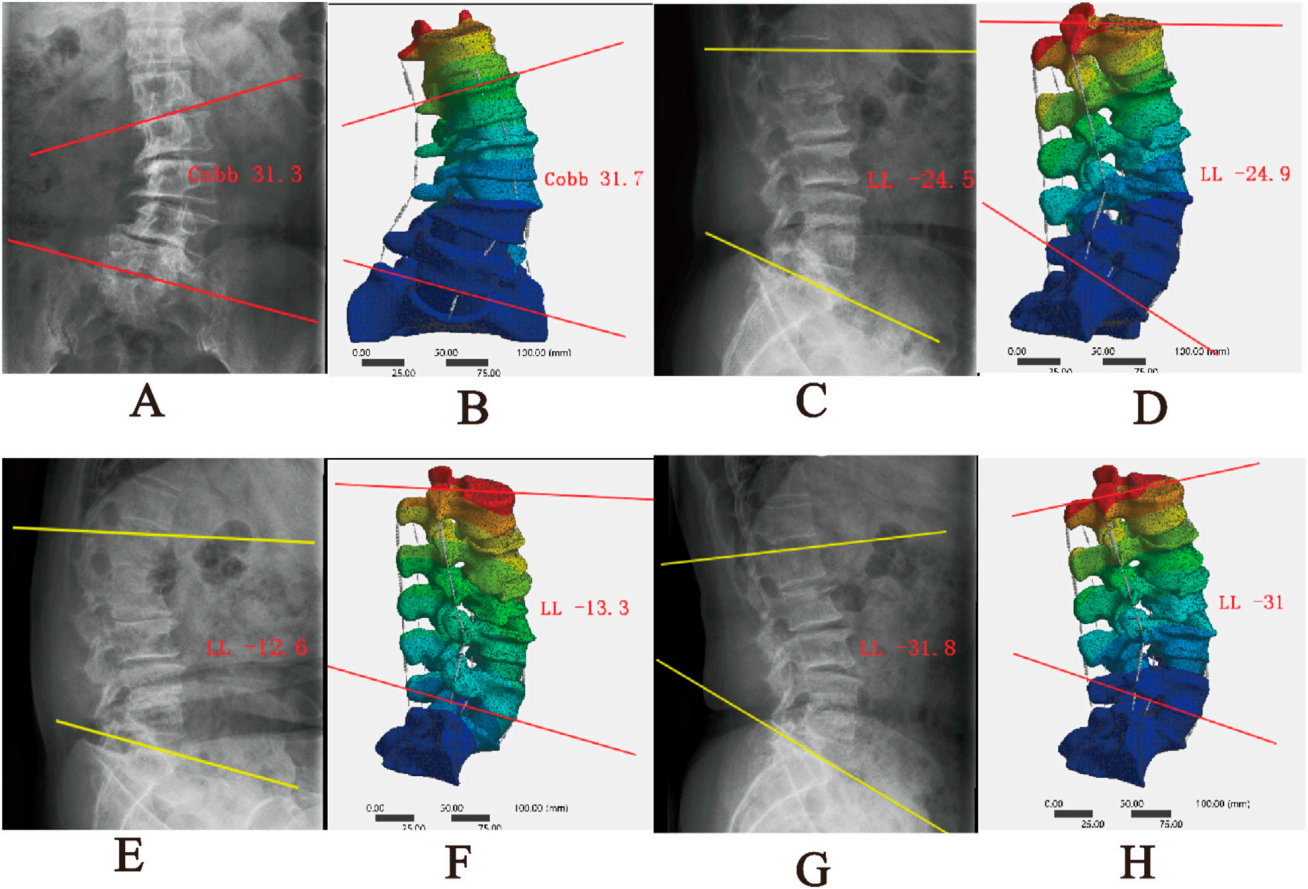
Comparison between the DLS model and actual radiographs; **(A,B)** Anteroposterior view; **(C,D)** Lateral view; **(E,F)** Flexion; **(G,H)** Extension.

The comparison between the DLS three-dimensional finite element model and the patient’s preoperative lumbar spine anteroposterior and lateral X-rays showed differences in Cobb angle and LL of −0.2° and −0.4°, respectively. When compared with the patient’s preoperative spinal flexion-extension X-rays, the differences in LL were −0.7° and 0.8°, respectively. These results indicate that the DLS model exhibits good consistency in geometric morphology with the actual X-rays (Figure 6; Table 3).

**TABLE 3:** Geometric morphology with the actual X-rays (°).

Parameter	Cobb	LL	Flexion LL	Extension LL
X-rays	20.8	−24.5	−12.6	−31.8
Finite element model	21	−24.9	−13.3	−31
Errors	−0.2	−0.4	−0.7	0.8

#### 3.3.2 Centroid measurement results

**FIGURE 7:**
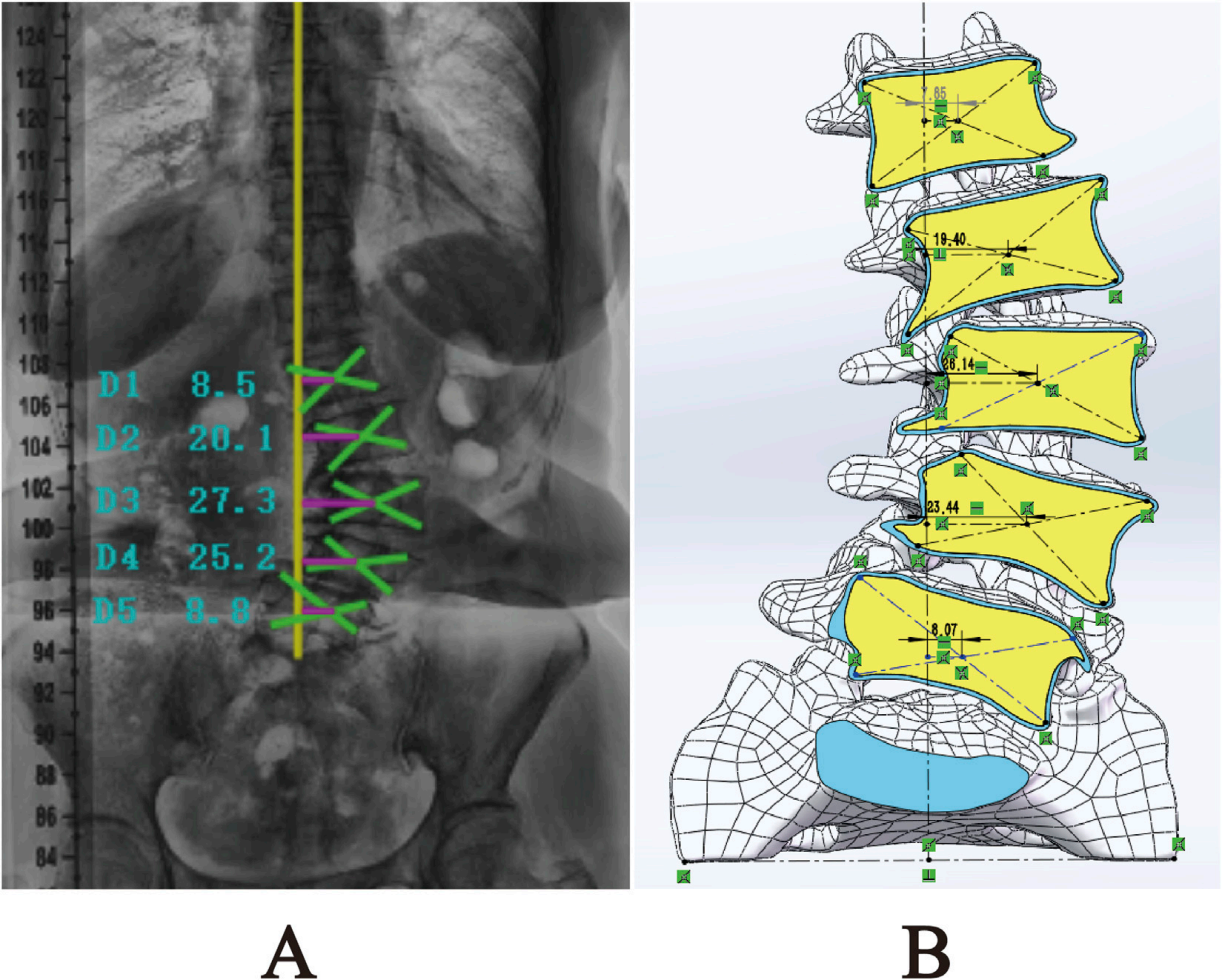
Comparison of L1-L5 to CVSL distance between the 3D finite element DLS model and preoperative anteroposterior radiographs: **(A)** Actual radiograph; **(B)** DLS model.

After measuring and comparing the distances from the center of each vertebral body L1-L5 to the CVSL on the preoperative anteroposterior X-rays with those in the DLS three-dimensional finite element model, the maximum distance difference was found to be 1.76 mm for L4-CVSL, and the minimum distance difference was 0.65 mm for L1-CVSL. This indicates that the DLS three-dimensional finite element model is in substantial agreement with the actual X-rays (Figure 7; Table 4).

**TABLE 4:** Centroid measurement results (mm).

Segment	X-rays	Finite element model	Errors
L1-CVSL	8.5	7.85	0.65
L2-CVSL	20.1	19.40	0.7
L3-CVSL	27.3	28.14	−0.84
L4-CVSL	25.2	23.44	1.76
L5-CVSL	8.8	8.07	−0.73

#### 3.3.3 ROM

**FIGURE 8:**
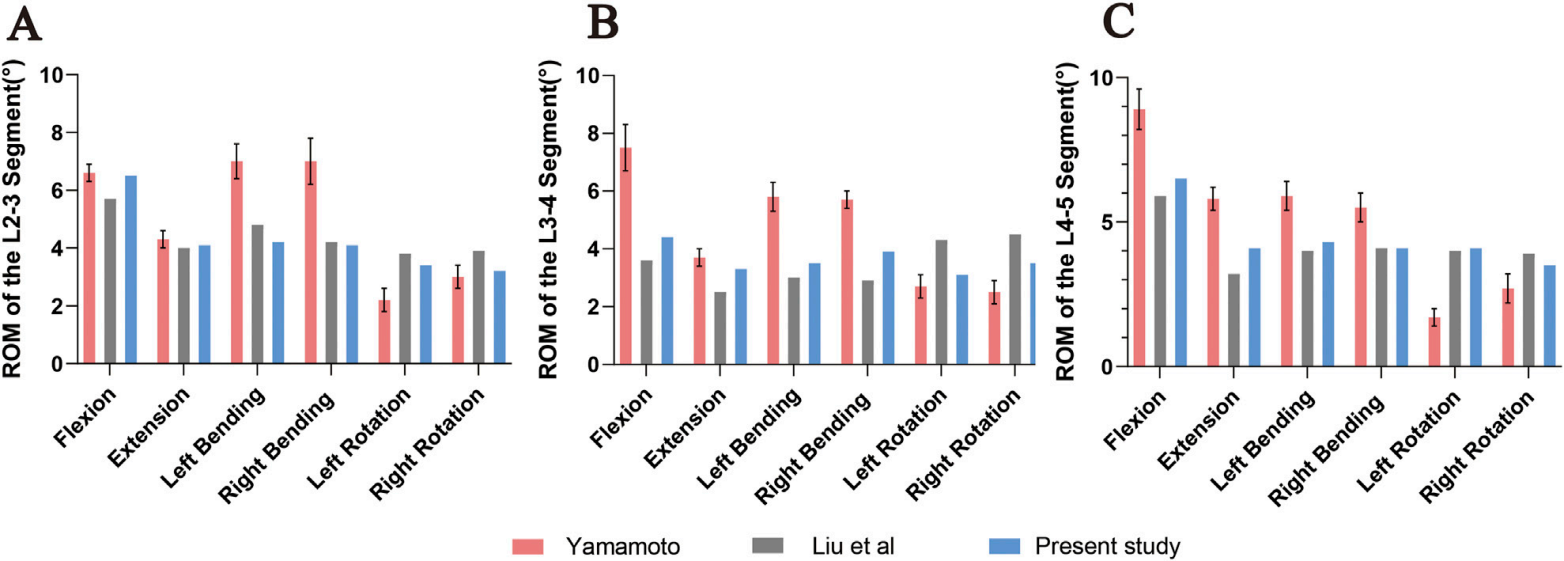
Comparison of ROM at L2-3 **(A)**, L3-4 **(B)**, and L4-5 **(C)** segments under flexion, extension, left/right lateral bending, and left/right axial rotation between the DLS model and previous studies. Error bars represent standard deviation.

The 3D finite element model of DLS established in this study calculated the ROM of each segment (L2-3, L3-4, L4-5) under the aforementioned load, torque, and constraint conditions. The results were compared with the *in vitro* experimental data from Yamamoto et al. (1989) and the finite element analysis results from Liu et al. (2017). It was found that the results obtained in this study were lower than those from Yamamoto et al.’s *in vitro* experiments, but were similar to the finite element analysis results from Liu et al. (Figure 8). The trend of ROM changes in this study was generally consistent with the results reported in previous literature. Therefore, it can be concluded that the 3D finite element model of DLS established in this study has high simulation accuracy and strong reliability, and can be used for further finite element simulation studies.

## 4 Discussion

Orthopedic biomechanics is a significant branch within the field of orthopedics, integrating mechanical principles with clinical practice to provide a solid theoretical foundation for disease diagnosis, treatment planning, and prognosis evaluation. Traditional research methods, such as cadaver dissection and animal experiments, have numerous limitations when studying DLS, including limited specimen availability, modeling difficulties, and the inability to accurately simulate dynamic mechanical behaviors under human physiological conditions. In contrast, the three-dimensional finite element model, as an advanced numerical simulation tool, can precisely replicate the geometric structure, material properties, and mechanical behaviors of the lumbar spine, offering a novel perspective for in-depth exploration of the therapeutic outcomes of DLS surgical treatments.

Currently, there are relatively few reports on the establishment and validation of finite element models for DLS. Kim et al. (2009) established two models, the scoliosis model and the scoliosis with rotation model, based on the CT data of a patient without spinal deformity by displacing and rotating the vertebrae. However, these models could not accurately reflect the pathological characteristics of DLS. In 2015, Zheng et al. (2015) successfully constructed a three-dimensional finite element model of the T12-S1 segment based on CT data from a DLS patient. This model not only accurately reflects the pathological characteristics of DLS patients but also comprehensively considers factors such as degenerated intervertebral discs, facet joints, and osteoporosis. However, given the complexity and diversity of DLS classification, although the model selected by Zheng et al. (2015) in their study is categorized as DLS, its Cobb angle is only 10.8°, and the degree of degeneration is relatively mild. Therefore, this model still struggles to accurately reflect the complex pathological characteristics present in most patients with degenerative scoliosis, and its universal applicability is somewhat limited. Compared with the mild degenerative scoliosis model constructed by Zheng et al., the present Lenke–Silva type IV reconstruction exhibits a Cobb angle of 21°, pronounced coronal imbalance, marked vertebral rotation, and advanced disc degeneration. Specifically, the annulus fibrosus elastic modulus was elevated to 8.4 MPa, indicative of moderate-to-severe degeneration. In contrast, Zheng’s model retained a nearly intact disc with an annulus modulus of only 4.2 MPa. Moreover, our model revealed markedly asymmetric stress concentration within the facet joints (peak von Mises stress = 5.78 MPa), the location of which corresponded precisely to the annular tear identified on pre-operative MRI. These findings underscore the enhanced capacity of a moderate-to-severe degenerative model to reproduce pathological biomechanical behaviours. Consequently, the current reconstruction is more representative of the typical DLS patient encountered clinically and is better suited for pre-operative planning and complication prediction. Furthermore, although the geometric features of the DLS model established by Zheng et al. (2015) are highly consistent with the actual X-rays of patients, this validation is primarily based on static geometric parameters (Cobb angle and LL). However, the biomechanical behavior of the spine is a dynamic process, and verification based solely on static geometric parameters may not fully reflect the accuracy of the model under dynamic conditions. In this study, stress loading verification was conducted on the model to simulate physiological activities of the human body in different directions (flexion, extension, left lateral flexion, right lateral flexion, left rotation, and right rotation). The ROM predicted for the L2–L5 segments in the present study was systematically lower than the values reported by Yamamoto et al. in their cadaveric experiments. This divergence is attributable to four principal factors: (1) Boundary-condition mismatch—the sacrum was fully constrained in our model, whereas residual visco-elasticity of the remaining soft tissues in cadaveric specimens may have artificially elevated the recorded ROM. (2) Material idealisation—all structures were assumed to be linear-elastic, neglecting the non-linear and visco-elastic behaviour of soft tissues, which likely led to an underestimation of the physiological ROM. (3) Degenerative pathology—the model was derived from a Lenke–Silva type IV degenerative scoliosis patient; advanced disc degeneration, osteophyte formation, and ligament calcification intrinsically reduce segmental mobility. (4) Absence of muscular contributions—active muscle contraction was not simulated, whereas residual muscle tension in the cadaveric tests may have influenced the observed motion. Collectively, the discrepancy between the computational and experimental data is biomechanically plausible within the context of a pathological spine and highlights the need for future models that integrate non-linear material properties and active musculature.

Mesh convergence plays a crucial role in finite element analysis, ensuring that the analysis results can accurately approximate the true solution, avoiding erroneous conclusions due to unreasonable mesh division, thereby providing a reliable basis for engineering design and decision-making (Ayturk and Puttlitz, 2011). Moreover, through grid convergence analysis, it is possible to reasonably control grid density while meeting accuracy requirements, thereby balancing computational precision and cost, and avoiding unnecessary waste of computational resources. Research indicates that the grid convergence of lumbar spine finite element models is significantly influenced by the direction of motion, with axial rotation being the most sensitive to grid resolution (Ayturk and Puttlitz, 2011). Given the clinical significance of stress analysis in lumbar spine finite element analysis, this study employs Von Mises stress to evaluate mesh convergence (Xu et al., 2017a). The study found that the cartilaginous endplate in the DLS model is the tissue most sensitive to mesh resolution, a result consistent with the research by Ayturk and Puttlitz (2011) and Xu et al. (2017a). The trend of percentage differences in Von Mises stress across different meshes for other structures in the DLS model is also similar to the findings of Xu’s study. The mesh-dependent variation in von Mises stress observed across different spinal tissues in the present study closely parallels the findings reported by Xu et al. (2017) for healthy lumbar finite-element models. Specifically: (1) the cartilaginous endplate exhibited the highest sensitivity to mesh refinement, with a 13.4% difference between Mesh 1 and Mesh 3 in our study compared with 41.15% in Xu’s work, confirming that the endplate is the most mesh-sensitive structure; (2) the intervertebral disc was comparatively insensitive, showing 6.8% variation herein versus 4.96% in Xu’s study, indicating consistent trends; and (3) cortical and cancellous bone demonstrated intermediate sensitivity, both exhibiting progressive stress convergence with increasing mesh density. Moreover, Xu et al. likewise employed von Mises stress as the convergence criterion and identified axial rotation as the loading direction most sensitive to mesh resolution, aligning perfectly with our methodological approach. These concordances demonstrate that, despite modeling a pathological (Lenke–Silva type IV DLS) spine, our mesh-sensitivity behavior remains consistent with established biomechanical modeling norms, thereby reinforcing the stability and reliability of the stress responses predicted by the model.

The three-dimensional finite element model of DLS constructed in this study, through the integration of multimodal imaging data and biomechanical validation, provides a crucial theoretical tool for clinical research. In the anatomical dimension, the error range between the model’s geometric parameters and the X-ray measurements (Cobb angle and LL error <1°, vertebral body center offset error <1.76 mm) confirms its morphological fidelity, laying a reliable foundation for simulating the three-dimensional mechanical effects of different surgical treatment strategies. In biomechanical evaluation, by incorporating individualized bone density parameters of patients, the model can quantitatively assess the stress distribution of pedicle screws and the risk of cage subsidence, providing a biomechanical basis for surgical planning. Additionally, the model can be utilized for the design and optimization of medical devices. By simulating the interaction between medical devices and human tissues, it evaluates their mechanical performance and clinical efficacy, offering a scientific foundation for the research and development of medical devices. In terms of model validation, this study verified the accuracy of anatomical morphology through comparison of imaging geometric parameters. It confirmed the reliability of the DLS model using vertebral center offset and ROM assessment. The results showed that the ROM of the model in flexion and extension movements differed by less than 15% from the *in vitro* experimental data of Yamamoto et al. (1989), and by less than 8% from the finite element study of Liu et al. (2017). Such deviation ranges are considered acceptable in the field of biomechanical modeling. The ROM values predicted by the current model differ by less than 15% from the cadaveric data of Yamamoto et al. and by less than 8% from the finite-element results of Liu et al.; both discrepancies fall within the tolerance limits widely accepted in spinal biomechanics. Ayturk and Puttlitz (2011) explicitly state that finite-element predictions exhibiting ≤ ±15% deviation from experimental data, together with consistent trend agreement, are indicative of sound biomechanical validity. Moreover, they recommend axial rotation as the most conservative loading mode for mesh-convergence assessment, a criterion that was also employed in the present study. Consequently, the current reconstruction is more representative of the typical DLS patient encountered clinically and may serve as a potential tool for pre-operative planning and complication-risk assessment; its clinical predictive accuracy must still be confirmed through multicentre, multi-patient prospective studies. It is particularly noteworthy that the model successfully replicated the high stress concentration phenomenon in the degenerated intervertebral disc (Von Mises stress reaching 5.78 MPa), which highly coincides with the location of the annulus fibrosus tear shown in the MRI of DLS patients, revealing the key mechanical mechanism in the degeneration process.

However, this study still faces several technical bottlenecks that need to be overcome. Firstly, the model in this study primarily simulates structures such as vertebrae, intervertebral discs, articular cartilage, and ligaments, but it has not yet incorporated the dynamic mechanical simulation of the lumbar and back muscle groups. The absence of active contraction mechanisms in core muscles like the multifidus and erector spinae may affect the accuracy of dynamic simulations of scoliosis compensation mechanisms. Secondly, due to the difficulty in obtaining cadaver specimens, model validation mainly relies on comparisons with historical research data. This indirect validation approach may obscure errors arising from individual differences. Additionally, the construction and analysis of the finite element model depend on various assumptions and simplifications, such as the homogenization of material properties and the linear simplification of nonlinear soft tissue characteristics. Although these assumptions are within a reasonable range, they may still have some impact on the results. Future research will focus on refining the three-dimensional finite element model of DLS by incorporating muscle tissue dynamics and optimizing dynamic load simulations, with validation through biomechanical experiments. The enhanced model will enable systematic evaluation of different surgical approaches’ biomechanical effects while facilitating the development of patient-specific models for improved preoperative planning and rehabilitation strategies. Further investigations will examine long-term postoperative biomechanical changes and their clinical implications through longitudinal follow-up studies. These advancements aim to strengthen the model’s clinical relevance, providing comprehensive theoretical foundations for DLS diagnosis, treatment optimization, and rehabilitation protocols.

## 5 Conclusion

This study successfully developed and validated a reliable three-dimensional finite element model of Lenke-Silva type IV DLS, which demonstrated geometric accuracy, mesh convergence, and biomechanical validity through comprehensive morphological and stress-loading analyses, effectively reproducing pathological anatomical features and simulating dynamic mechanical behaviors under complex loads to support surgical optimization, implant evaluation, and complication prediction in DLS research. The present reconstruction is based on a single patient, and future work will enrol a larger cohort to establish population-level validity and integrate active muscle dynamics to further enhance individualised surgical planning.

## Data Availability

The original contributions presented in the study are included in the article/Supplementary Material, further inquiries can be directed to the corresponding authors.

## Supplementary material

SUPPLEMENTARY FIGURE S1 Optimization of the 3D model and cortical/cancellous bone surface fitting process in Geomagic Wrap 2021 software. **(A)** Raw model; **(B)** Mesh Doctor; **(C)** Remove spikes; **(D)** Remove features; **(E)** Smoothing and noise reduction; **(F)** Detect contour lines; **(G)** Generate contour lines; **(H)** Create surface patches; **(I)** Construct grid; **(J)** Fit surfaces; **(K)** Inward offset (extract cancellous bone model); **(L)** Trim posterior structures and patch holes; **(M)** Smoothing; **(N)** Construct grid; **(O)** Fit surfaces.

SUPPLEMENTARY FIGURE S2 Modeling of articular cartilage and complete model construction.

SUPPLEMENTARY FIGURE S3 Command script **(A)** and corresponding results **(B)**.

SUPPLEMENTARY FIGURE S4 Mesh sensitivity analysis of the DLS 3D finite element model.
