# Supplementary figures and images for: Establishment and validation of a three-dimensional finite element model for degenerative lumbar scoliosis
Source: Front Bioeng Biotechnol. 2025 Nov 27;13:1669961. doi: 10.3389/fbioe.2025.1669961 (PMC12695814; doi:10.3389/fbioe.2025.1669961)

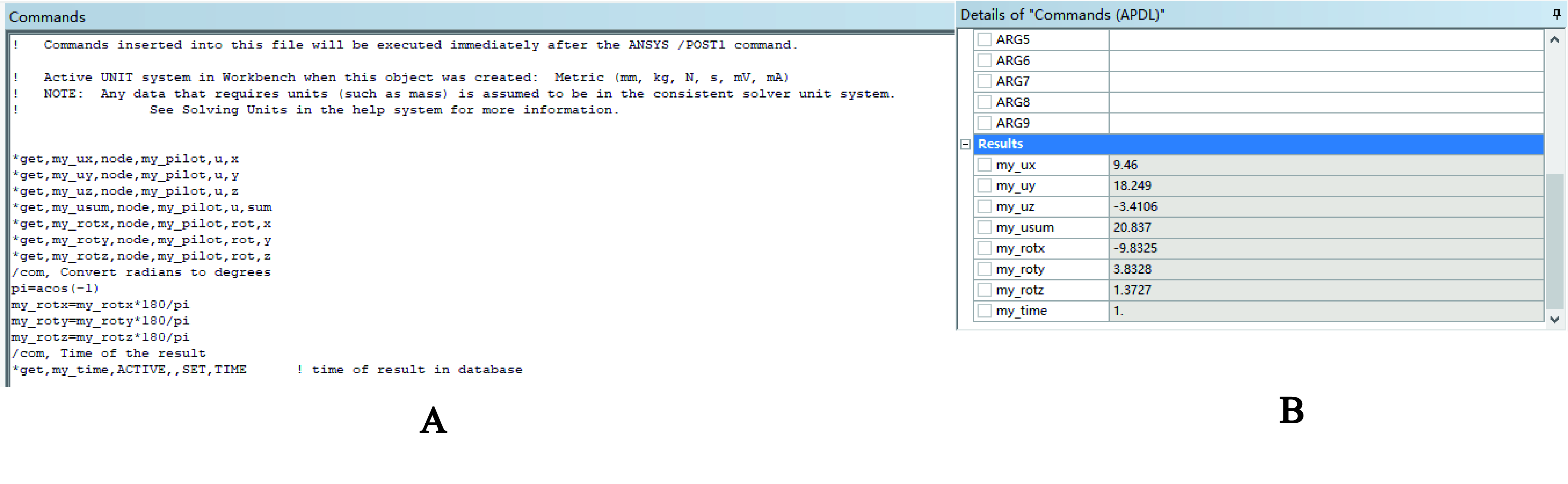

Supplement: Supplementary file 1 [file Image3.tif]

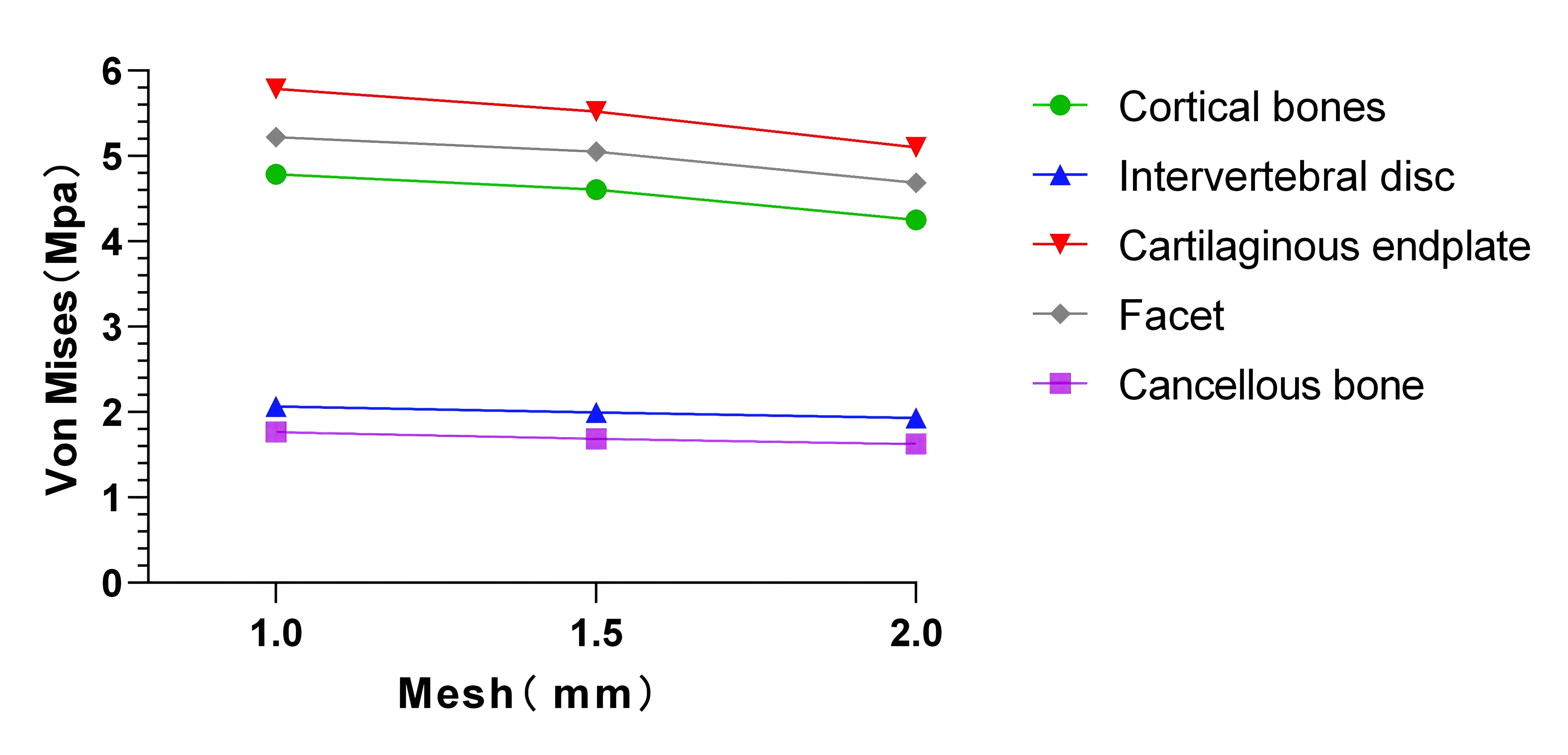

Supplement: Supplementary file 2 [file Image4.tif]

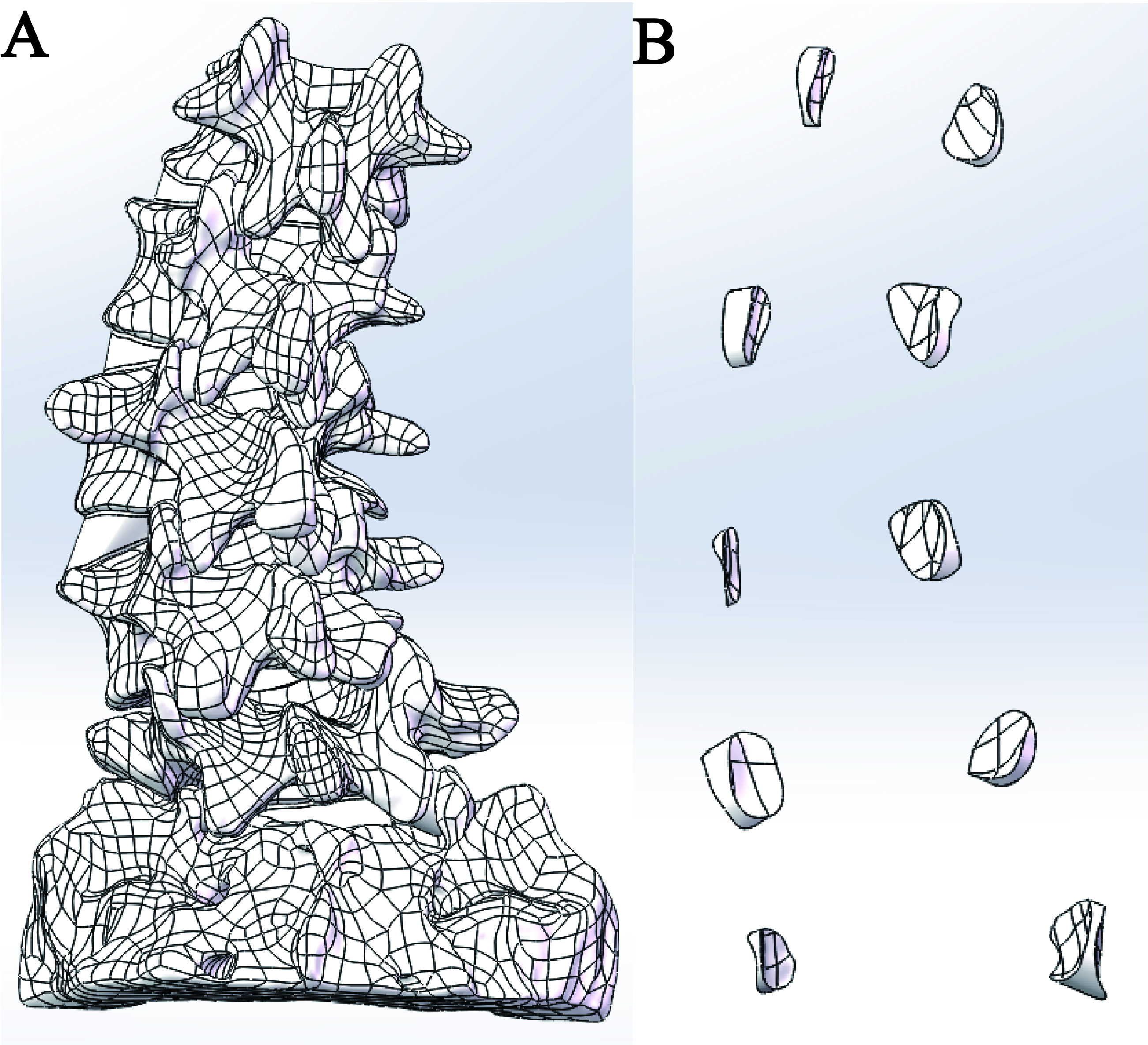

Supplement: Supplementary file 3 [file Image2.tif]

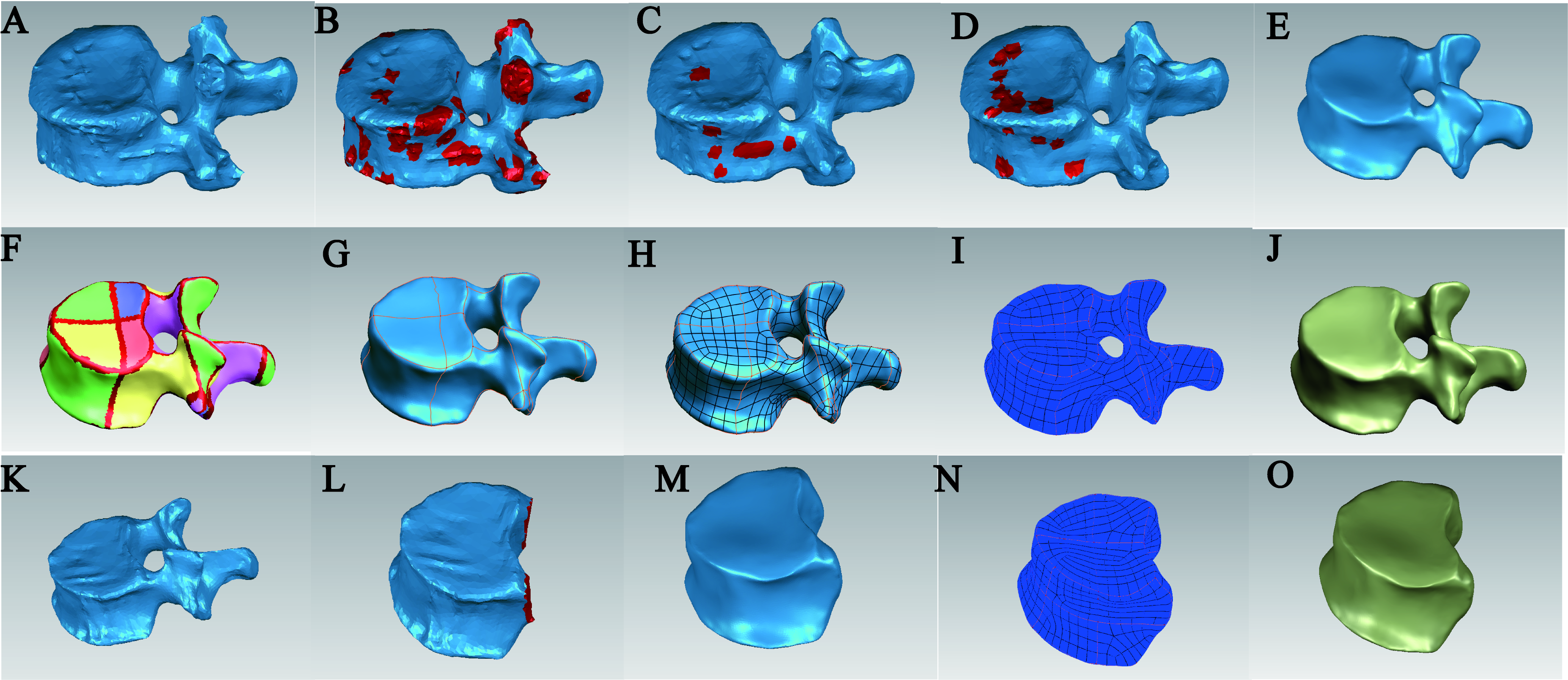

Supplement: Supplementary file 4 [file Image1.tif]
